# A Double WAP Domain-Containing Protein Es-DWD1 from *Eriocheir* *sinensis* Exhibits Antimicrobial and Proteinase Inhibitory Activities

**DOI:** 10.1371/journal.pone.0073563

**Published:** 2013-08-13

**Authors:** Shuang Li, Xing-Kun Jin, Xiao-Nv Guo, Ai-Qing Yu, Min-Hao Wu, Shang-Jian Tan, You-Ting Zhu, Wei-Wei Li, Qun Wang

**Affiliations:** School of Life Science, East China Normal University, Shanghai, China; National Cancer Institute, NIH, United States of America

## Abstract

Whey acidic proteins (WAP) belong to a large gene family of antibacterial peptides, which are critical in the host immune response against microbial invasion. The common feature of these proteins is a single WAP domain maintained by at least one four-disulfide core (4-DSC) structure rich in cysteine residues. In this study, a double WAP domain (DWD)-containing protein, Es-DWD1, was first cloned from the Chinese mitten crab ( *Eriocheir* *sinensis* ). The full-length *Es-DWD1*cDNA was 1193 bp, including a 411 bp open reading frame (ORF) encoding 136 amino acids with a signal peptide of 22 amino acids in the N-terminus. A comparison with other reported invertebrate and vertebrate sequences revealed the presence of WAP domains characteristic of WAP superfamilies. As determined by quantitative real-time RT-PCR, *Es-DWD1* transcripts were ubiquitously expressed in all tissues, but it was up-regulated in hemocytes post-challenge with pathogen-associated molecular patterns (PAMPs). The mature recombinant Es-DWD1 (rEs-DWD1) protein exhibited different binding activities to bacteria and fungus. Moreover, rEs-DWD1 could exert agglutination activities against *Bacillus subtilis* and *Pichia* *pastoris* and demonstrated inhibitory activities against the growth of *Staphylococcus aureus, Aeromonas hydrophila* and *P* *. pastoris* . Furthermore, rEs-DWD1 showed a specific protease inhibitory activity in *B. subtilis*. Coating of rEs-DWD1 onto agarose beads enhanced encapsulation of the beads by crab hemocytes. Collectively, the results suggest that Es-DWD1 is a double WAP domain containing protein with antimicrobial and proteinase inhibitory activities, which play significant roles in the immunity of crustaceans.

## Introduction

Antimicrobial peptides are critical in the initial host defense system of many animal species [1] and exist in a wide range of organisms, including microorganisms, protozoa, invertebrates, vertebrates and plants [2]. Thousands of antimicrobial peptides have been reported, but few have been found in the decapods of crustaceans [3]. Among the reported antimicrobial peptides, proteins containing whey acidic protein (WAP) domains, initially characterized as a milk protein, have been found in several species of both vertebrates and invertebrates [3,4]. The WAP domain is characterized by a ‘four-disulﬁde-core’ (4-DSC) motif comprised of approximately 50 amino acids with eight highly conserved cysteine residues [3,5]. The WAP domain is not exclusive to WAP proteins but is found in numerous other proteins, where it may be present as multiple domains [6].

The WAP-containing proteins that have been isolated and characterized in crustaceans are crustins, which contain single and double WAP domains (DWD). The common structures of crustins include a signal peptide sequence, a glycine-rich domain at the N-terminus and a conserved sequence of cysteine residues at the C-terminus. All reported crustins are classiﬁed into several types which differ in the amino acid sequences between the signal peptides and the WAP domains. Type I and II crustins characterized in several species of crustaceans have been shown to be abundant and to exhibit antimicrobial activity mainly against Gram-positive bacteria [3]. Type III crustins are WAP domain-containing proteins from decapods lacking both of the glycine-rich domain of crustin II molecules and the cysteine-rich region present in crustin I and crustin II [3]. Recently, two studies described crustin-like proteins in shrimp that have two WAP domains, a signal sequence and no other motifs [7,8]. These are named DWD proteins, but can be tentatively considered as type IV crustins, at least until additional information is available [9]. Proteins containing the WAP domain exist in many species with a variety of biological functions [10–13]. The WAP domain is capable of inhibiting certain proteinases and exhibits antimicrobial activity [14]. It is well documented that double DWD proteins such as Kazal [15–17], pacifastin [18], serpin [19] and alpha 2-macroglobulin are proteinase inhibitors which can counteract the proteinases of pathogens [20–24]. For instance, Fc-DWD from *Fenneropenaeus* *chinensis* binds both Gram-negative and Gram-positive bacteria and exhibits proteinase inhibitory activities [25]. Mj-DWD from shrimp *Marsupenaeus* *japonicus* plays an important role in the host defense against white spot syndrome virus (WSSV) infection, possibly through its protease inhibitory activity [8]. The synthesis of DWD proteins has been shown to be upregulated by inoculation with WSSV in *M* *. japonicus* and by injecting the heat-killed *Vibrio* *alginolyticus* in *Litopenaeusvannamei* in hemocytes during an early phase of infection [8,26].

The Chinese mitten crab *Eriocheir* *sinensis* , a freshwater crustacean reproducing in seawater, has signiﬁcant advantages over its smaller arthropod counterparts in studies of innate immune mechanisms [27]. *E. sinensis* aquaculture is a rapidly developing industry in China, with a peak yield of 40 million tons in 2005 [28]. However, various diseases caused by bacteria, viruses and richettsia-like organisms have resulted in catastrophic losses and have become a major constraint for the development of the crab aquaculture industry [29–31]. Therefore, in order to understand the innate immune system of crabs and their defense mechanisms, we investigated the function of *E. sinensis* DWD1 (Es-DWD1) through gene cloning, profiling tissue-speciﬁc expression, determining the mRNA tissue-dependent expression patterns following challenge with LPS (lipopolysaccharides), PG(peptidoglycans) and Glu (β-1,3-glucans). Moreover, we purified the recombinant Es-DWD1 (rEs-DWD1) protein and analyzed its antimicrobial and proteinase inhibitory activities.

## Materials and Methods

### Sample preparation and immune challenge of animals

Adult Chinese mitten crabs (n = 200; 100 ± 12 g wet weight) were purchased from the Tongchuan Aquatic Product Market in Shanghai, China and cultured in filtered aerated freshwater at 20–25°C. Before being dissected, crabs were lightly anesthetized by placement in an ice bath. Tissues of hemocytes, heart, hepatopancreas, gill, stomach, muscle, intestine, testis, thoracic ganglia and brain were collected, snap frozen in liquid nitrogen and stored at −80° C before nucleic acid analysis. For cloning and expression analysis, tissues from 10 individuals were pooled and ground with a mortar and pestle prior to extraction. Hemolymph was collected from the last pair of walking legs from each crab by using a syringe and then quickly added to an anticoagulant solution (0.1 M glucose, 30 mM citrate, 26 mM citric acid, 0.14 M NaCl, 10 mM EDTA) [32] at a 1:1 ratio. The hemolymph was then separated by centrifugation at 800 × *g*, 4° C, and hemocytes were collected.

One hundred and sixty healthy crabs were divided equally into four groups for PAMP stimulation. Three experimental crab groups were injected into the arthrodial membrane of the last pair of walking legs with approximately 100 µl of PG (peptidoglycans) from *Staphylococcus aureus* (Sigma-Aldrich, St. Louis, MO, USA), 100 µl of LPS (lipopolysaccharides) from *Escherichia coli* (Sigma-Aldrich) and 100 µl of β-1,3-glucans (Glu from *Saccharomyces cerevisiae*, Sigma-Aldrich) resuspended (500 ug/mL) in *E. sinensis* saline (ESS, 0.2 M NaCl, 5.4 mM KCl, 10.0 mM CaCl_2_, 2.6 mM MgCl_2_, 2.0 mM NaHCO_3_; pH 7.4) [32,33]. Crabs of the control group were injected with 100 µl ESS (pH 7.4). More than five crabs were selected randomly at each time interval of 0 (as blank control), 2, 6, 12 and 24 h after injection with each type of PAMP. Hemocytes were obtained as described above and after the addition of 1 mL Trizol reagent (Invitrogen, Carlsbad, CA, USA) for subsequent RNA extraction, the samples were stored at -80° C.

### Total RNA extraction and first-strand cDNA synthesis

Total RNA was extracted from the above-mentioned tissues sampled from section “Sample preparation and immune challenge of animals” using Trizol reagent (RNA Extraction Kit, Invitrogen) according to the manufacturer’s instructions. The total RNA concentration and quality were estimated using spectrophotometry at an absorbance of 260 nm and agarose gel electrophoresis, respectively. For quantitative real-time RT-PCR (qRT-PCR) expression analysis, total RNA (4 μg) was reverse transcribed using the PrimeScript™ Real-time PCR Kit (TaKaRa, Shiga, Japan).

For full-length cDNA cloning, total RNA (5 μg) isolated from testis and hemocytes tissue were reverse transcribed respectively using the SMARTer™ RACE cDNA Amplification kit (Clontech, Mountain View, CA, USA).

### Cloning of full-length *Es-DWD1* cDNA

The *Es-DWD1* partial cDNA sequence was extended using 5’ and 3’ RACE (SMARTer™ RACE cDNA Amplification kit, Clontech) and two gene-specific primers (Table S1) based on the initial sequence from our previously constructed *Eriocheir Sinensis* transcriptome profiles of testis [34] and hemocytes (unpublished). The 3’ RACE PCR reaction was carried out in a total volume of 50 μl containing 2.5 μl (800 ng/μl) of the first-strand cDNA reaction as a template, 5 μl of 10X Advantage 2 PCR buffer, 1 μl of 10 mM dNTPs, 5 μl (10 μM) gene-specific primer (Es-DWD1-3’RACE, Table S1), 1 μl of Universal Primer A Mix (UPM; Clontech), 34.5 μl of sterile deionized water and 1 U 50X Advantage 2 polymerase mix (Clontech). For the 5’ RACE, UPM was used as forward primers in PCR reactions in conjunction with the reverse gene-specific primers (Es-DWD1-5’RACE, Table S1). PCR amplification conditions for both the 3’ and 5’ RACE were as follows: 5 cycles at 94° C for 30 s, 72° C for 3 min; 5 cycles at 94° C for 30 s, 70° C for 30 s, and 72° C for 3 min; 20 cycles at 94° C for 30 s, 68° C for 30 s, and 72° C for 3 min. Resulting data were analyzed using the CFX Manager^TM^ software (version 1.0).

### Sequence analysis and phylogenetic analysis

Full-length *Es-DWD1*cDNAs and deduced amino acid sequences were compared against sequences from other representative vertebrates and invertebrates reported in the NCBI GenBank using the BLAST program (http://blast.ncbi.nlm.nih.gov). These analyses were completed by multiple sequence alignment using ClustalX and ClustalW2 (http://www.ebiac.uk/Tools/msa/clustalw2/). An unrooted neighbor-joining phylogenetic tree was constructed with MEGA 5.0. The homologous conserved domains and signal peptides were identified by the SMART (Simple Modular Architecture Reseach Tool, http://smartembl-heidelberg.de.) program. Three-dimensional protein structure modeling of the predicted amino acid sequence of Es-DWD1 was carried out using the SWISS-MODEL server.

### Transcriptional analysis by qRT-PCR

qRT-PCR was performed by SYBR^®^ Premix Ex Taq^TM^ (TaKaRa) with *Es-DWD1* gene-specific primer pairs (Table S1). PCR conditions were as follows: 30 cycles at 94° C for 30 s, 58° C for 30 s, and 72° C for 1 min. Internal control PCR reactions for *β-actin* were performed in a separate tube as described above with the exception of an alternative gene-specific primer pair (Table S1), which was designed based upon a cloned *E. sinensis β-actin* [35] cDNA fragment to produce a 276 bp amplicon. All qRT-PCR reactions were completed in triplicate using independently extracted RNA. For the transcriptional analysis of *Es-DWD1* in various tissues and after the immune challenge, the PCR template was obtained as described in section “Sample preparation and immune challenge of animals”. The *Es-DWD1* relative expression levels were calculated by the 2^−ΔΔCt^ comparative *C*_*T*_ method [36].

### Expression and purification of rEs-DWD1 in *E. coli*

The cDNA fragment encoding the mature peptide of *Es-DWD1* was amplified by the primers Es-DWD1-F (5’-CCGG A A T T CCAGGGCACAAGGGGAGGACT-3’) and Es-DWD1-R (5’-CCGC T C G A GCTACAATGGCTCCGAACACT-3’), which included the *Eco*RI and *Xho*I restriction sites (underlined), respectively. The amplified products were cloned into the pET32a vector (Novagen, Darmstadt, Germany) with a His-tag and then transformed into *E. coli* BL21 (DE3) competent cells (Tiangen, China) for expression.

The bacteria were grown at 37° C in Luria-Bertani (LB) medium. When the OD_600_ value reached 0.6, isopropyl β-D-1-thiogalactopyranoside (IPTG) was added into the culture media (1mM final concentration) to induce recombinant protein expression. After culturing at 37^°^ C overnight, bacteria pellets were collected after centrifugation at 6000 × *g* for 5 min and resuspended in 8 ml of guanosine lysis buffer. The lysate prepared under hybrid conditions was loaded onto a purification column for His•Bind resin chromatography according to the manufacturer’s instructions (Invitrogen). Briefly, lysate solution was used to keep the resin suspended for 30–60 min. The target protein with the His-tag was added to the purification column to bind with the resin. The resin was collected by low speed centrifugation (800 × *g*) and then washed with 8 ml of Native Wash Buffer four times. The protein sample was eluted with 8–12 ml of Native Elution Buffer and separated electrophoretically on a reducing 12% SDS-PAGE gel and visualized with Coomassie Brilliant Blue R250. The concentrations of purified rEs-DWD1 proteins were quantified by the Bradford method.

### Western blotting assay

The binding activity of recombinant proteins towards three Gram-negative bacteria (*Vibrio parahaemolyticus*, *Aeromonas hydrophila and E. coli*), two Gram-positive bacteria (*B. subtilis* and S. *aureus*) and one fungus ( *P* *. pastoris* ) was tested by Western blotting. Bacteria and fungus were cultured overnight in 5 ml LB and YPD (Yeast Peptone Dextrose) medium, respectively, and then collected by centrifugation (6000 × *g*) for 5 min. The bacteria and fungus were washed three times with TBS and then thoroughly resuspended in 2 ml of TBS (OD_600_ approximately 1.0). Purified rEs-DWD1 (0.1 mg/ml; 500 μl) was incubated with 500 μl of the microorganism suspension (2 × 10^7^ cells/ml) in TBS on a rotator for 1 h at 37^°^ C. The mixed microorganisms were collected by centrifugation (6000 × *g*, 5 min), washed four times with TBS and then lysed with 10% SDS (20 μl). The lysates were separated by 12% SDS-PAGE. Recombinant Es-DWD1 proteins were detected using specific His-tag antibodies by Western blot [37].

### Antimicrobial activity assays

The bacteria growth inhibitory activity of the rEs-DWD1 protein was tested against *S. aureus*, *A. hydrophila* and *P* *. pastoris* . These three microorganisms were cultured overnight until the OD_600_ value reached 1.0. Different final concentrations of the rEs-DWD1 protein were added to the *S. aureus*, *A. hydrophila* and *P* *. pastoris* cultures and incubated with aeration at 200 rpm. The OD_600_ value was measured every 2 h.

The antimicrobial activity of the rEs-DWD1 protein against *E. coli* and *S. aureus* was tested on petri dishes. The different strains were coated on a petri dish and incubated overnight. Then, the single colonies were inoculated into the corresponding medium and collected by centrifugation (6000 × *g*) for 10 min. The bacteria was resuspended in TBS (50 mM Tris-HCl, 100 mM NaCl, pH 7.5), mixed with warm nutrient agar (1.0%) and poured into a petri dish [38]. Perforex was used to produce 0.5-cm diameter pores in the agar. Equal concentrations of antibiotic, rTrx or recombinant rEs-DWD1 protein in 100 μl TBS was added to each pore. The microbes were incubated overnight at the suitable temperature. TBS and rTrx were added to the pores as controls.

### Microbial agglutination assay

The Gram-positive (*B. subtilis* and *S. aureus*), Gram-negative (*E. coli* and *Pseudomonas aeruginosa*) and fungus ( *P* *. pastoris* ) were used in bacterial agglutination assays. Approximately 1.5 ml of each strain of cultured microbes (OD_600_> 1) were centrifuged at 6000 × *g* for 10 min [39]. The pellets were washed and resuspended in TBS. Recombinant Es-DWD1(10 μl) and bacteria (20 μl) were mixed together and incubated at room temperature for ~2 h before observation of agglutination by bright light microscopy (Leica, DM 4000B). TBS and rTrx were used as controls.

### In vitro cellular adhesion assay

*In vitro* encapsulation was tested by His-tagged rEs-DWD1 (TBS and rTrx as controls) coated nickel agarose beads. The agarose beads were washed three times and equilibrated in TBS. Renatured His-tagged Es-DWD1 was added and incubated with the nickel agarose beads in a 1.5 mL tube at room temperature for 1 h. Protein-coated beads were washed four times with TBS (5 min each time) and finally resuspended in TBS. Hemocytes, which had been collected from each group of Chinese mitten crabs using a sterile syringe and simultaneously diluted in anticoagulant were added to the protein-coated beads (about 80–100 beads) and incubated at 18^°^ C for 2 h, 6 h and 24 h. The final reactions were observed by light microscopy (Leica, DM 4000B) [40].

### Protease inhibition assay

The disc diffusion technique was employed in the protease inhibition assay. A single *B. subtilis* colony was transferred with a sterile toothpick to a skim milk plate (1% agar, 1% skim milk) [8]. The *B. subtilis* colony was covered with a filter paper disc, and 10 μl of sterile-filtered rEs-DWD1 was added onto the paper disc. The skim milk plate was incubated at 28^°^ C overnight, and then the paper disc was removed carefully to observe the transparent zone. TBS and rTrx were used as negative controls.

Another protease inhibition assay was performed *in vitro* following the method described by Somprasong et al. (2006) [41]. Here, the inhibitory activity of rEs-DWD1 was assayed by incubation with subtilisin A (*Bacillus licheniformis*, Sigma). The reaction mixture consisted of 0.1 M Tris-HCl (pH 8.0) 1 μM N-succinyl-Ala–Ala–Pro–Phe-p-nitroanilide (AAPF, Sigma) for subtilisin A, subtilisin A (0.005 μM) and rEs-DWD1 (at 0, 3, 6, 9, 12 or 15 μM) in a ﬁnal volume of 100 μl. The mixture was pre-incubated for 15 min at 37^°^ C and then terminated by adding 50 μl of 50% (v/v) acetic acid. The formation of p-nitroaniline was monitored continuously by spectrophotometry at 405 nm. The remaining activity was calculated and plotted against the concentrations of inhibitor.

### Statistical analysis

Statistical analysis was performed using SPSS software (ver. 11.0). Data are represented as the mean ± standard error (S.E.). Statistical significance was determined by one-way ANOVA [42] and *post-hoc* Duncan multiple range tests. Differences were considered significant at *P* < 0.05 and extremely significant at *P* < 0.01.

## Results

### Cloning and characterization of *Es-DWD1*

**Figure 1:**
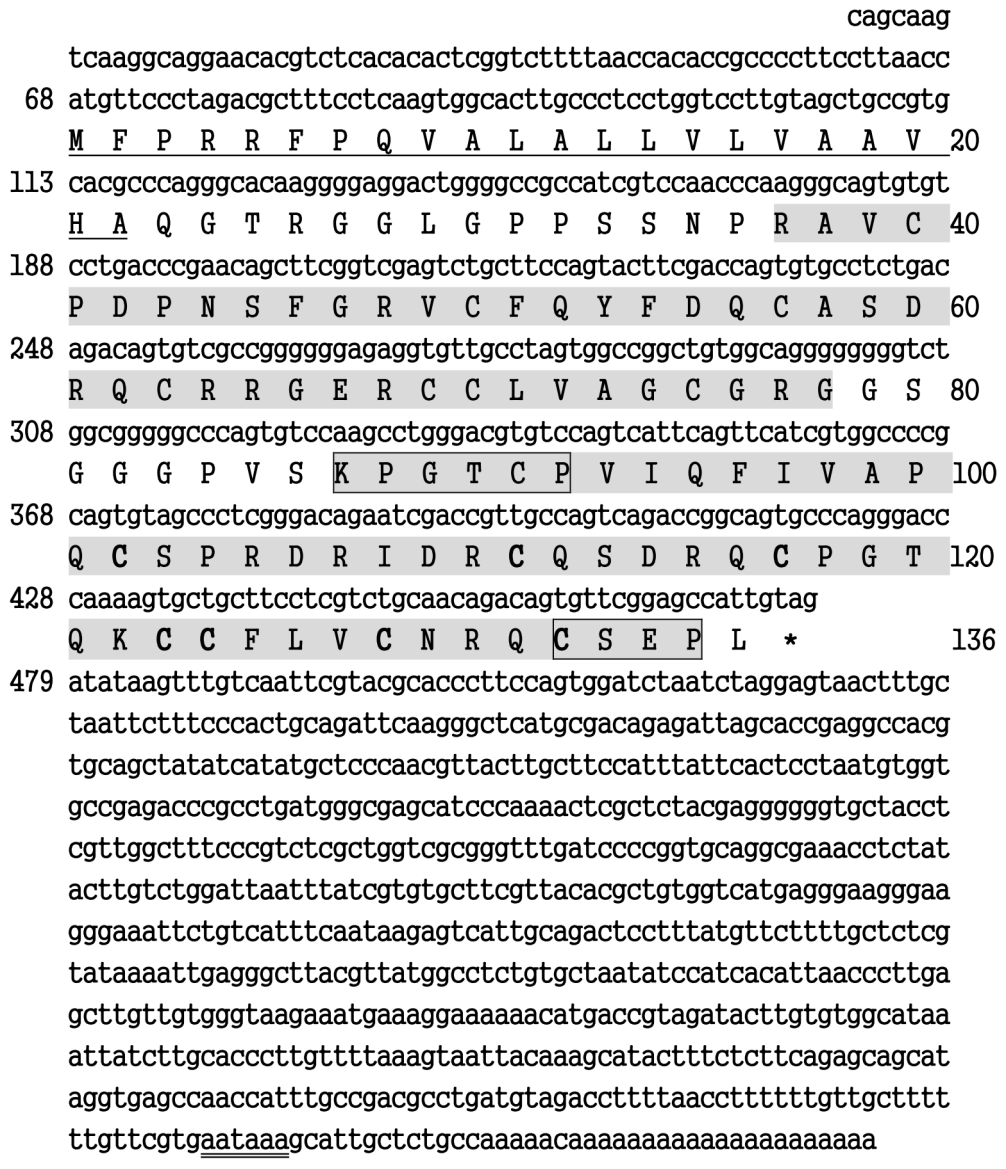
Nucleotide and deduced amino acid sequences of Es-DWD1. The nucleotide sequence is numbered from the first base at the 5’ end. The first methionine (M) is numbered as the first deduced amino acid. The WAP domains are highlighted, and the underlined sequence indicates the putative signal peptide (aa 1–22). The KxGxCP motif and the CxxP motif present in WAP domain II are boxed. The polyadenylation signal (AATAAA) is double underlined.

The full sequence of *Es-DWD1*, the *E. sinensis* double WAP domain cDNA obtained in this study (GenBank accession number: JX101865), was 1193 bp in length, containing a 411-bp ORF encoding a 136-amino acid protein, a 67-bp 5’ UTR and a 715-bp 3’ UTR (Figure 1). *Es-DWD1* was predicted to have a signal peptide of 22 amino acid residues and two WAP domains (domain I and domain II) by analysis of the deduced amino acid sequence using the SMART program (Figure 1). The conserved KxGxCP motif and CxxP motif were found in domain II of *Es-DWD1* (Figure 1).

A ClustalW2 alignment of *Es-DWD1* and homologous sequences obtained from a Protein Blast search shows the conserved cysteine residues believed to be characteristic of the WAP domain (Figure S1). The three-dimensional protein structure model predicted by SWISS-MODEL showed eight conserved cysteine residues in the WAP domain (Figure S2)

### Phylogenetic Analysis of Es-DWD1

**Figure 2:**
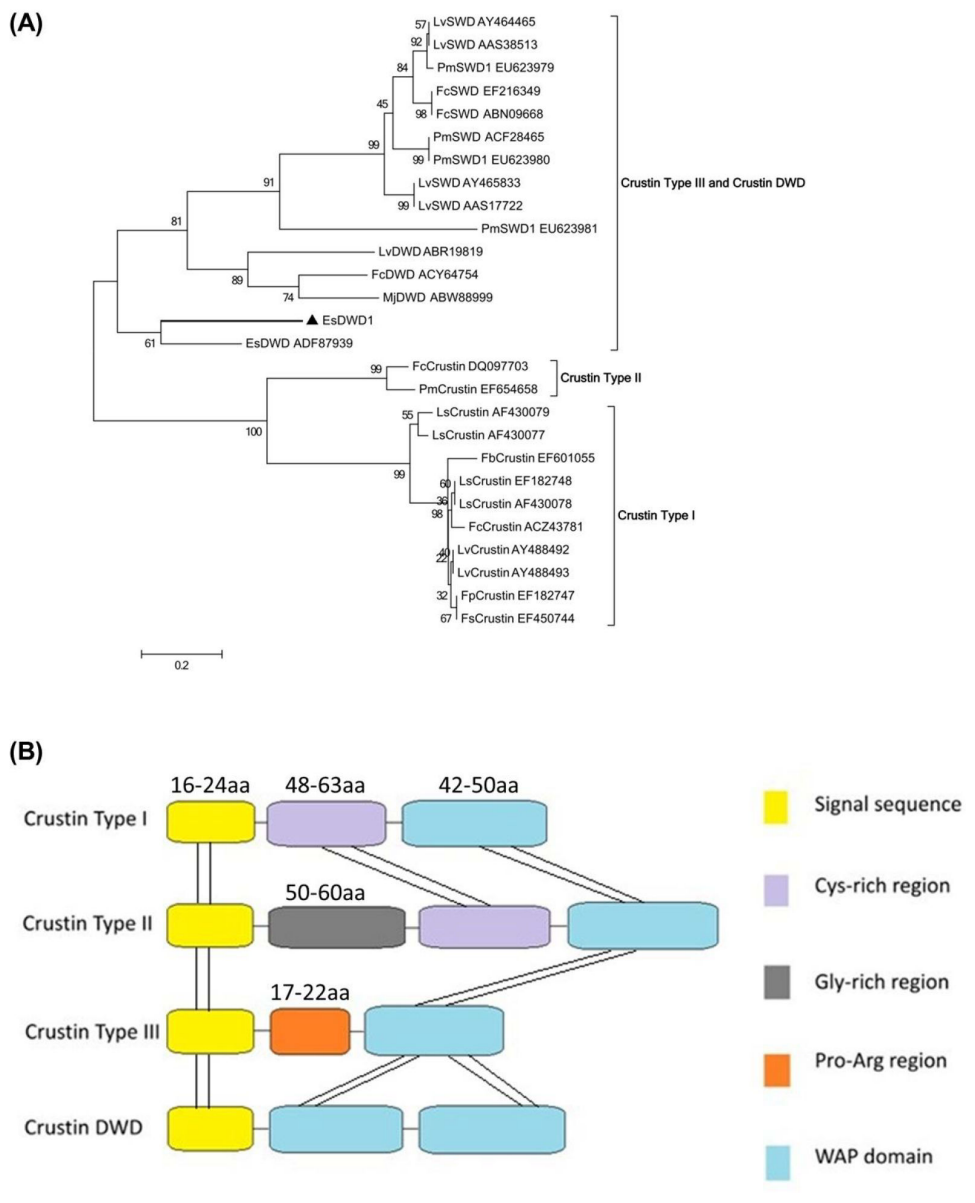
The neighbor-joining phylogenetic analysis and modular structures of WAP domain-containing proteins. (**A**) Unrooted neighbor-joining phylogenetic analysis of the Es-DWD1 (black triangle) amino acid sequences with WAP domain-containing proteins showing high similarity from a BLASTp homology search. The branches of *E*. *sinensis* DWD1 sequences are in bold. The tree was produced with MEGA 5.0 software. Three thousand bootstraps were carried out to check the repeatability of the results. Abbreviations: Lv, *L*. *vannamei*; Pm, P. *monodon*; Fc, *F*. *chinensis*; Es, *E*. *sinensis*; Mj. *M*. *japonicas*; Ls. *Litopenaeus setiferus*; Fb, *Farfantepenaeus brasiliensis*; Fp, *Farfantepenaeus paulensis*; Fs, *Farfantepenaeus subtilis*. (**B**) The positions of the WAP domain (blue) and other regions (Cys-rich region, Gly-rich region and Pro–Arg region; see color key within the figure) are indicted for each type of proteins. All proteins contain a signal peptide (yellow).

A phylogenetic analysis of the proteins using the neighbor-joining method showed that the WAP-containing proteins in crustaceans could be divided into four types (Figure 2B) and grouped into three different clusters (Figure 2A). These three clusters contained either 1) crustin type I, 2) crustin type II or 3) crustin type III and crustin DWD. *Es-DWD1* belonged to the crustin DWD group containing two WAP domains (Figure 2A).

### Tissue distribution of *Es-DWD1*

**Figure 3:**
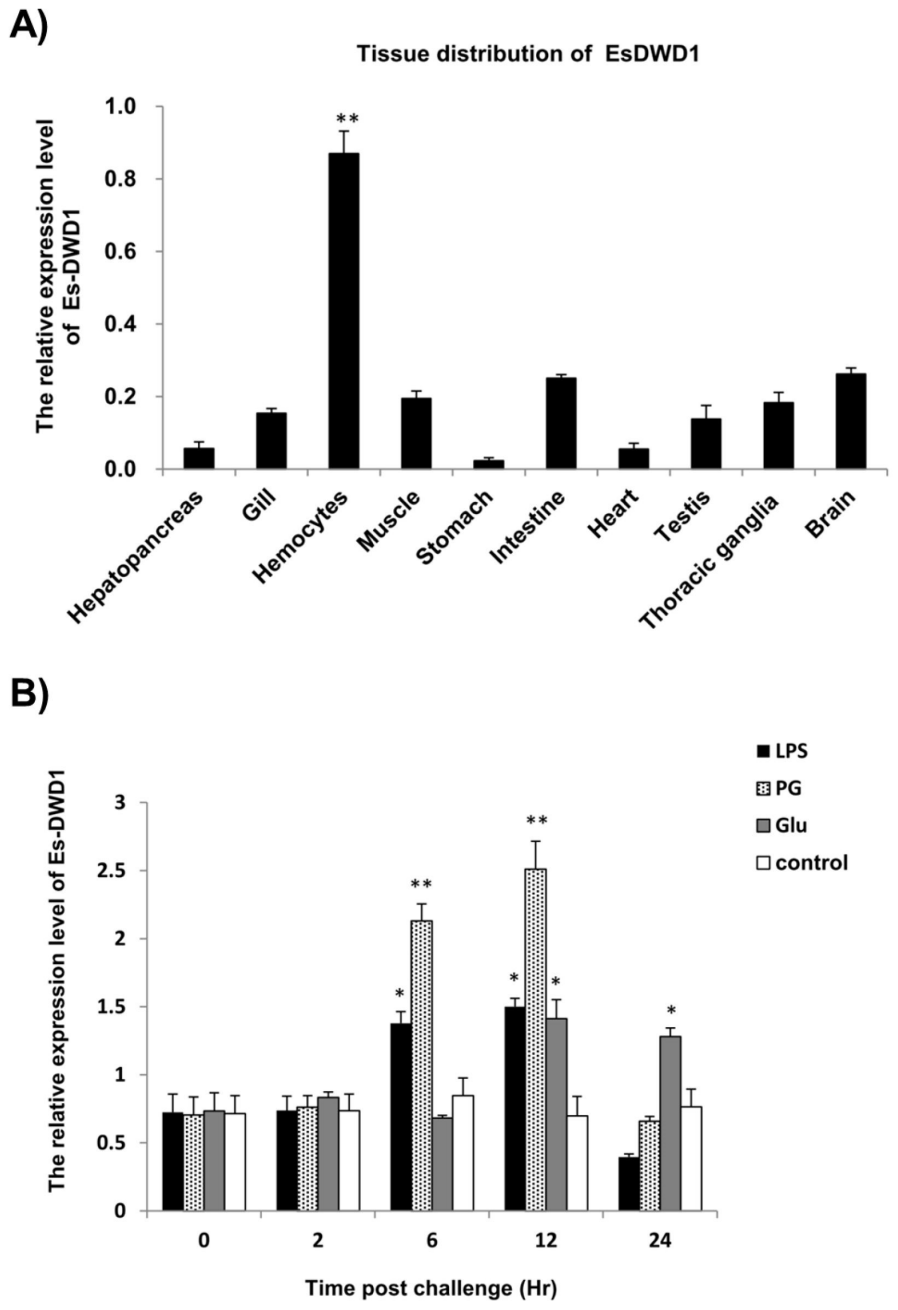
Tissue distribution and induction of *Es-DWD1* transcription after challenge with different PAMPs. (A) Analysis of *Es-DWD1* gene expression relative to *β-actin* in different Chinese mitten crab tissues by qRT-PCR. Tissues: hepatopancreas, gills, hemocytes, muscle, stomach, intestine, heart, testis, thoracic ganglia and brain. (B) *Es-DWD1* expression was significantly induced compared with the control (white bars) at the following time points: 6 h and 12 h after LPS challenge; 6 h, 12 h after PG challenge; 12 h, 24 h post Glu challenge. *P< 0.05; **P < 0.01.

As determined by qRT-PCR, *Es-DWD1* expression was widely observed in all detected tissues of *E. sinensis*, including hepatopancreas, gill, muscle, stomach, intestine, heart, testis, thoracic ganglia and brain, and it was dramatically abundant in immune-related tissues such as hemocytes (Figure 3A).

### Temporal expression of *Es-DWD1* after immune challenge with PAMPs

Based on the qRT-PCR results, Es-DWD1 expression levels in hemocytes were differentially inducible after challenge with Glu, LPS and PG. *Es-DWD1* expression was significantly higher than the blank control at 6 h and 12 h after the LPS injection (*P* < 0.05) (Figure 3B). *Es-DWD1* expression also was significantly upregulated at 6 h and 12 h after the PG injection (*P*< 0.01), peaking up to 5 times above the blank control after 12 h (Figure 3B), and at 12 h and 24 h after the Glu injection (*P*< 0.05) (Figure 3B). Meanwhile, control reactions with ESS yielded no significant variation in expression levels (Figure 3B, white bars).

### Expression and purification of rEs-DWD1

**Figure 4:**
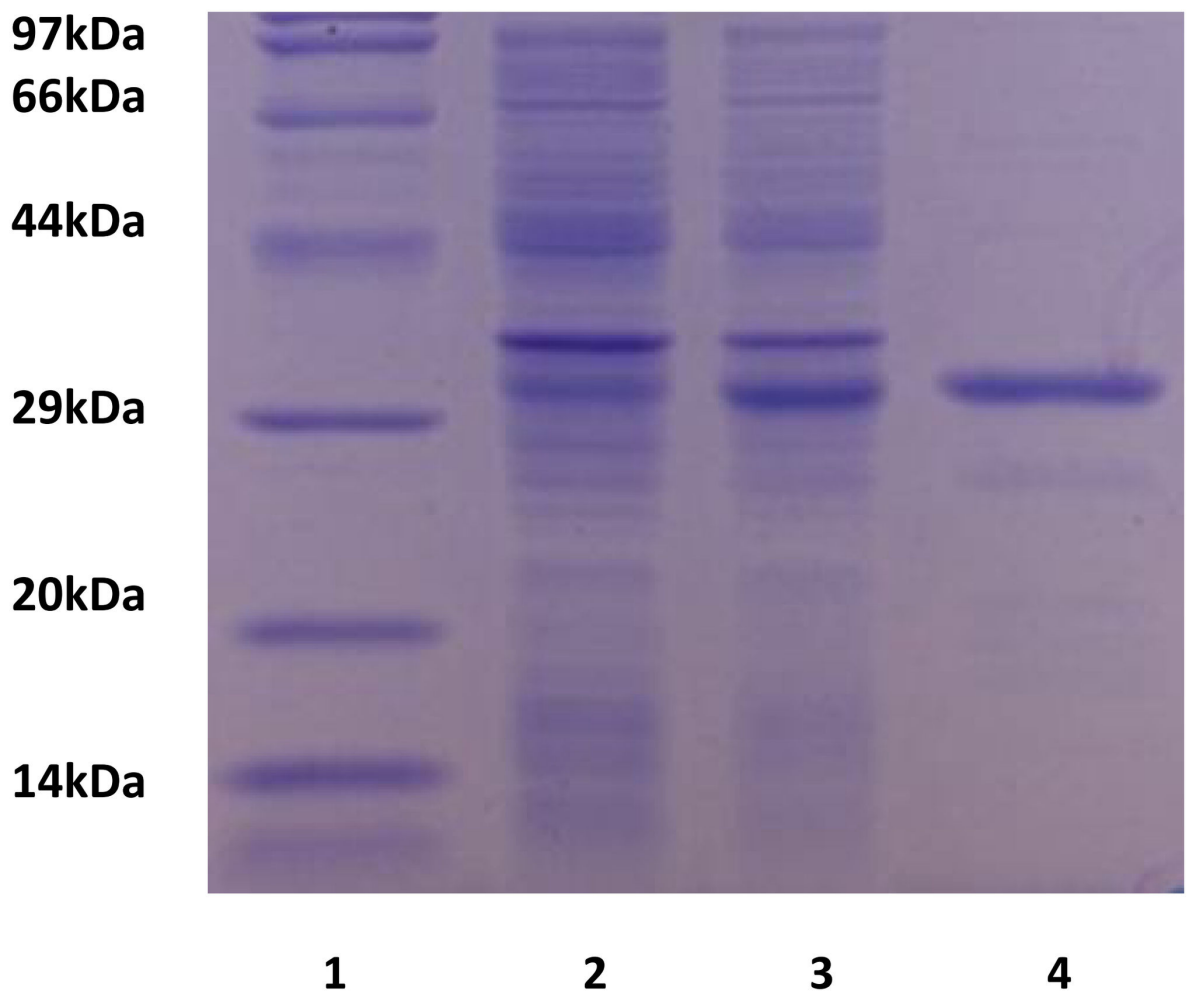
Analysis of expression and purification of rEs-DWD1 in *E*. ***coli* by SDS-PAGE**. Lane 1, protein standard; lane 2, total proteins of *E*. *coli* transformed with pET32a-Es-DWD1, without induction; lane 3, proteins of *E*. *coli* transformed with pET32a-Es-DWD1, induced with IPTG; lane 4, rEs-DWD1 purified by His•Bind Resin chromatography.

After IPTG induction of *E. coli* BL21 (DE3) cells transformed with the pET32a vector, the highly expressed recombinant protein with a molecular weight of ~32 kDa was detected as a distinct band in the whole cell lysate by SDS-PAGE analysis (Figure 4). Since the predicted molecular weight of rEs-DWD1 is ~14.6 kDa, the larger ~32 kDa detected reflected the fact that it was expressed as fusion protein with a Trx His-tag at the C-terminus. Insoluble recombinant proteins were extracted from the purified inclusion bodies with 8 M urea, renatured and purified using His•Bind Resin chromatography.

### Binding activity of rEs-DWD1 with microorganisms

**Figure 5:**
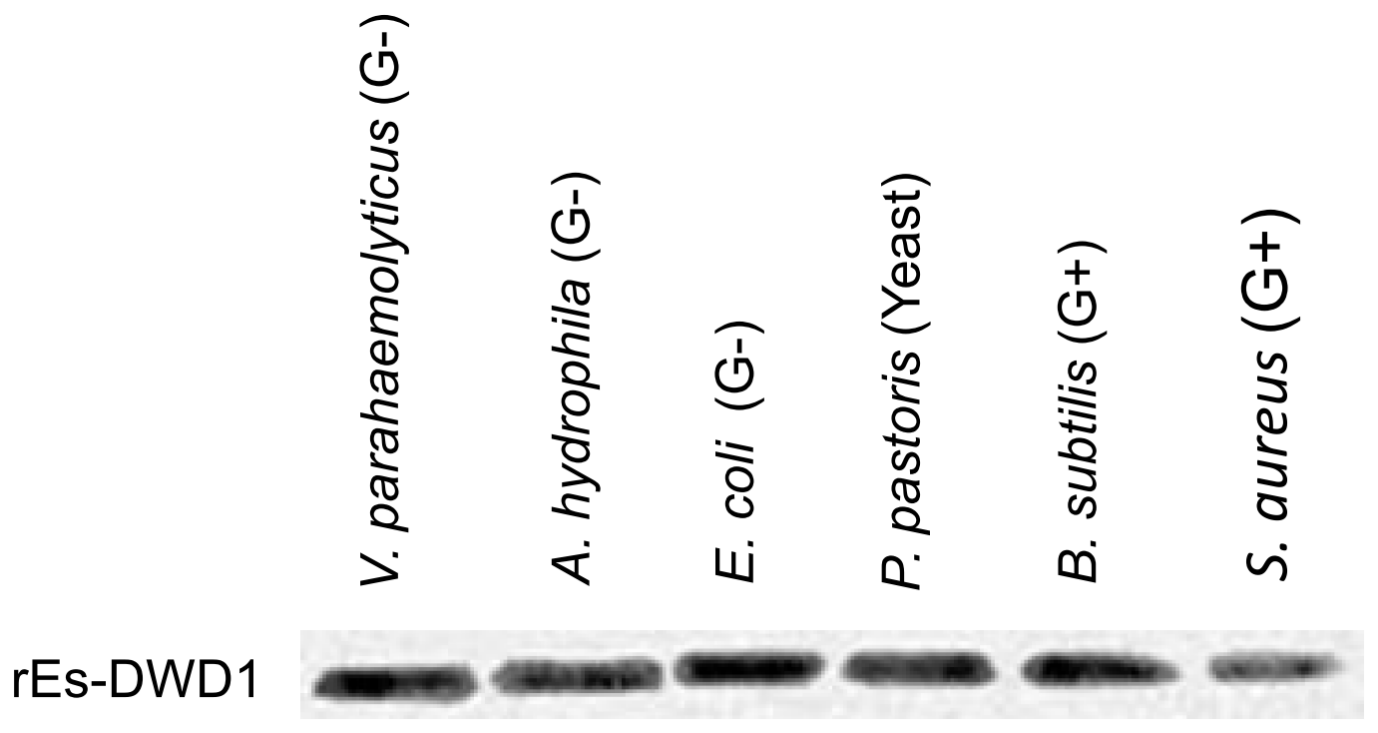
Western blot analysis of rEs-DWD1 binding with microorganisms. Recombinant proteins were incubated with three strains of Gram-negative bacteria (G^-^), two strains of Gram-positive bacteria (G^+^) and one fungus. Western blot assay was carried out using a His-tag antibody and developed using an ECL colorimetric substrate kit (CWbio, Beijing, China).

To test the ability of rEs-DWD1 to bind to microorganisms, we performed a direct binding assay by Western blotting (Figure 5). The results indicated that while rEs-DWD1 could bind to all six microorganisms tested (including three Gram-negative bacteria *V. parahaemolyticus*, *A. hydrophila and E. coli*; two Gram-positive bacteria *B. subtilis* and S. aureus, one fungus *P* *. pastoris* ), it bound to *E. coli* with the greatest efficiency. Thus, these results demonstrated distinct binding activities of the rEs-DWD1 protein.

### Antibacterial activity of rEs-DWD1

**Figure 6:**
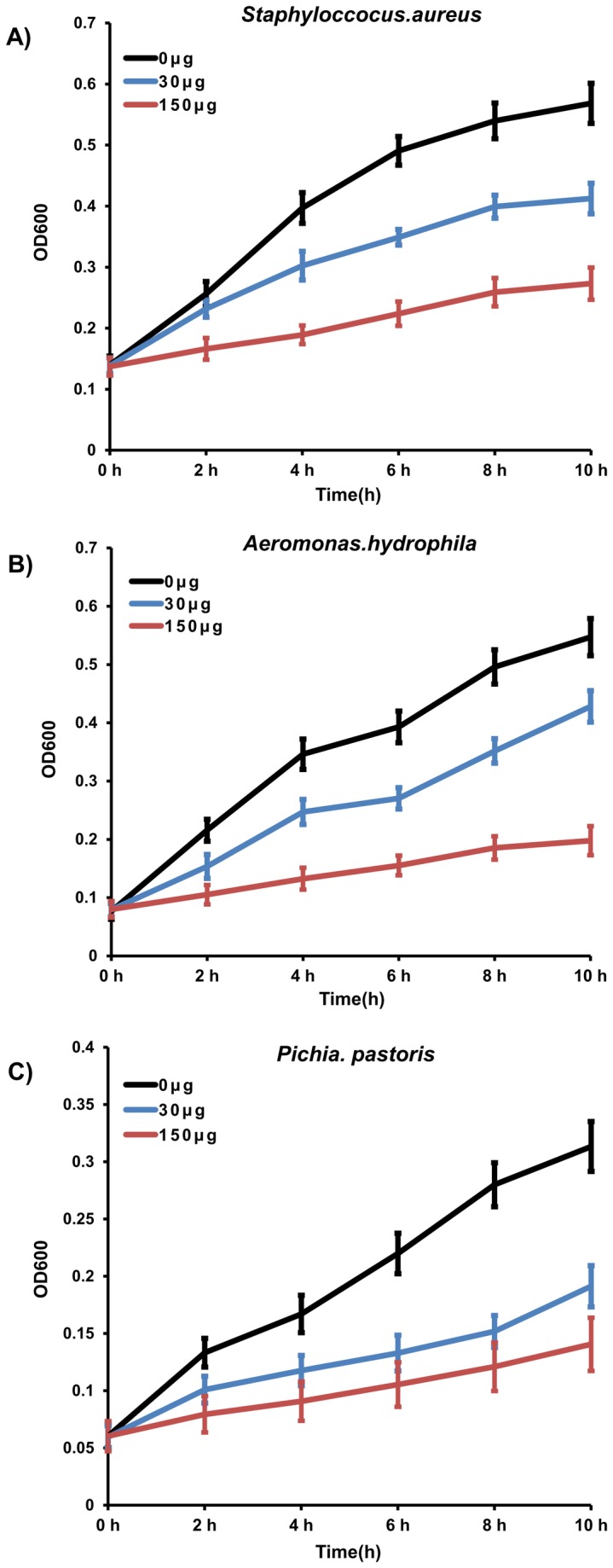
Growth suppression assays of rEs-DWD1 (0 μg, 30 μg and 150 μg/ml) against Gram-negative, Gram-positive bacteria and fungus. After starting the cultures, OD_600_values were measured every 2 h to construct the growth curves. (A) *S*. *aureus* G^+^ mixed with rEs-DWD1; (B) *A*. *hydrophila* G^-^ mixed with rEs-DWD1; (C) *P*. *pastoris* (fungus) mixed with rEs-DWD1. Data are presented as the mean ± SD of three independent cultures.

To determine the antimicrobial activity of the rEs-DWD1 protein, its inhibitory effects on the growth of microbes were examined. By Western blotting, the rEs-DWD1 protein exhibited the ability to bind to all six microbes mentioned above. Three of them were used in this assay. The antimicrobial activities of rEs-DWD1 against *S. aureus*, *A. hydrophila* and *P* *. pastoris* were dose-dependent, with 150 μg/ml rEs-DWD1 strongly suppressing microbial growth, while 30 μg/ml rEs-DWD1 mediated only weak suppression (Figure 6).

**Figure 7:**
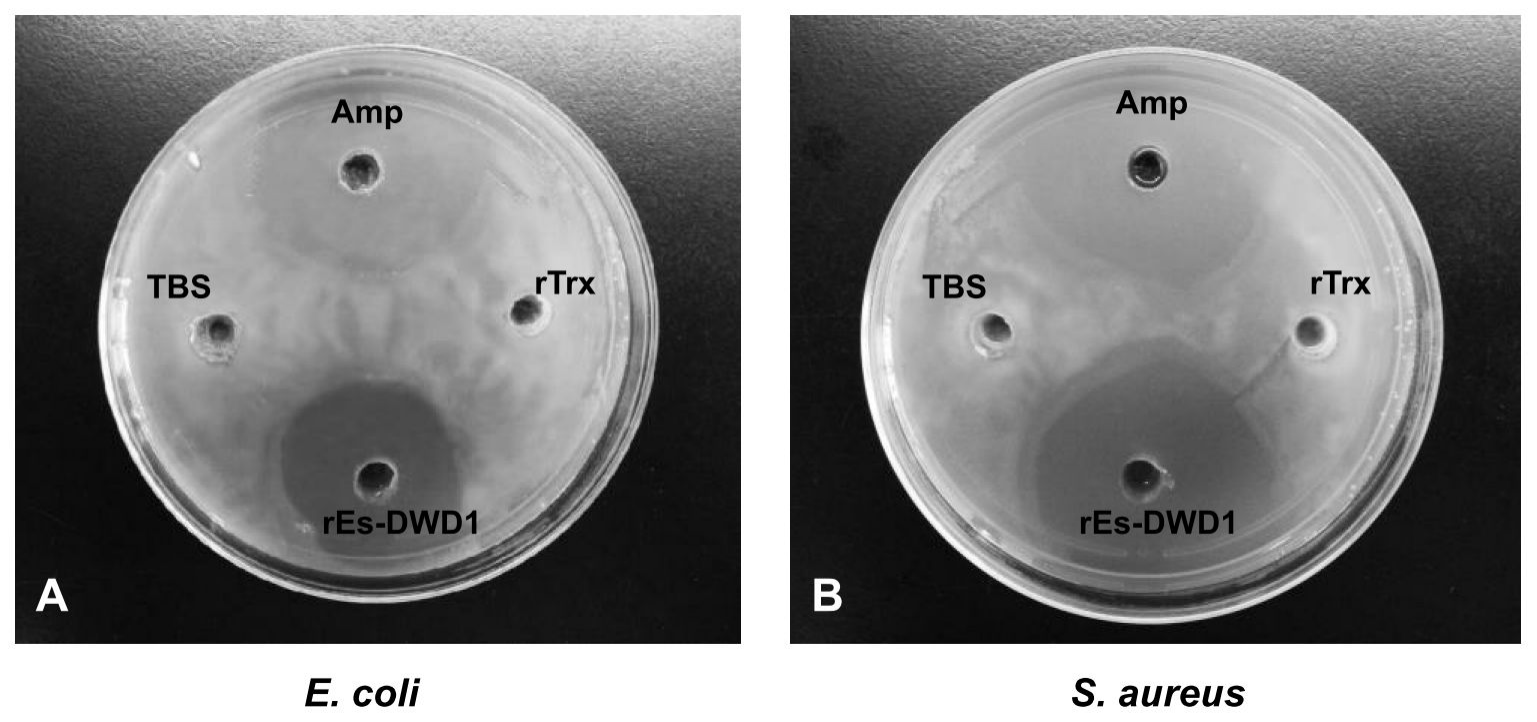
Anti-microbial activity of rEs-DWD1. Antibacterial activities against *E*. *coli* (A) and *S*. *aureus* (B) were assessed on petri dishes. rEs-DWD1 protein at the final concentration of 0.3 μg/μl or ampicillin (Amp) in 100 μl TBS was added to the pores of agar plates, mixed with microbes and then incubated at 37^°^ C for 16 h. TBS and rTrx used as control.

To test the antimicrobial activity of rEs-DWD1, its inhibitory effects on growth of *E. coli* and S. aureus were detected on agar plates. In contrast with the TBS and rTrx, transparent rings could be seen around the pores with rEs-DWD1, similar to those with the positive ampicillin control in the plates of *E. coli* and S. aureus (Figure 7)*.*

### Microorganism agglutination

**Figure 8:**
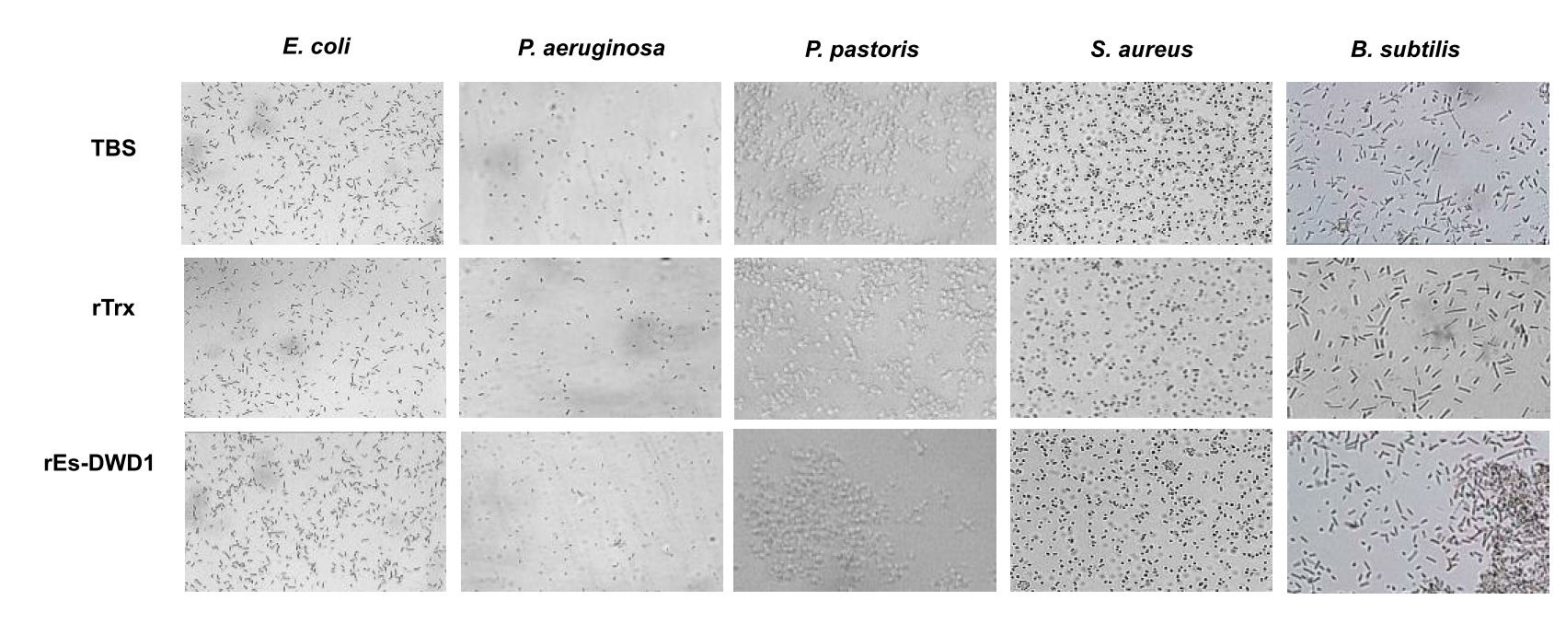
Bacterial agglutination activity assay. Gram-negative bacteria (*E*. *coli, P*. *aeruginosa*) Gram-positive bacteria (*S*. *aureus, B*. *subtilis*) or fungus (*P*. *pastoris*) were incubated with TBS, rTrx or with rEs-DWD1.

Bacteria and fungus were used to test the agglutination effects of rEs-DWD1 by binding to the surface of different microorganisms. Gram-positive bacteria (*B. subtilis* and *S. aureus*), Gram-negative bacteria (*E. coli*, and *P. aeruginosa*) and fungus ( *P* *. pastoris* ) were incubated with rEs-DWD1 protein. The results indicated that addition of rEs-DWD1 caused significant aggregation of *B. subtilis* and *P* *. pastoris* (Figure 8). Meanwhile, no obvious agglutination was observed when rEs-DWD1 was incubated with the other bacteria or the control group.

### In vitro cellular adhesion

**Figure 9:**
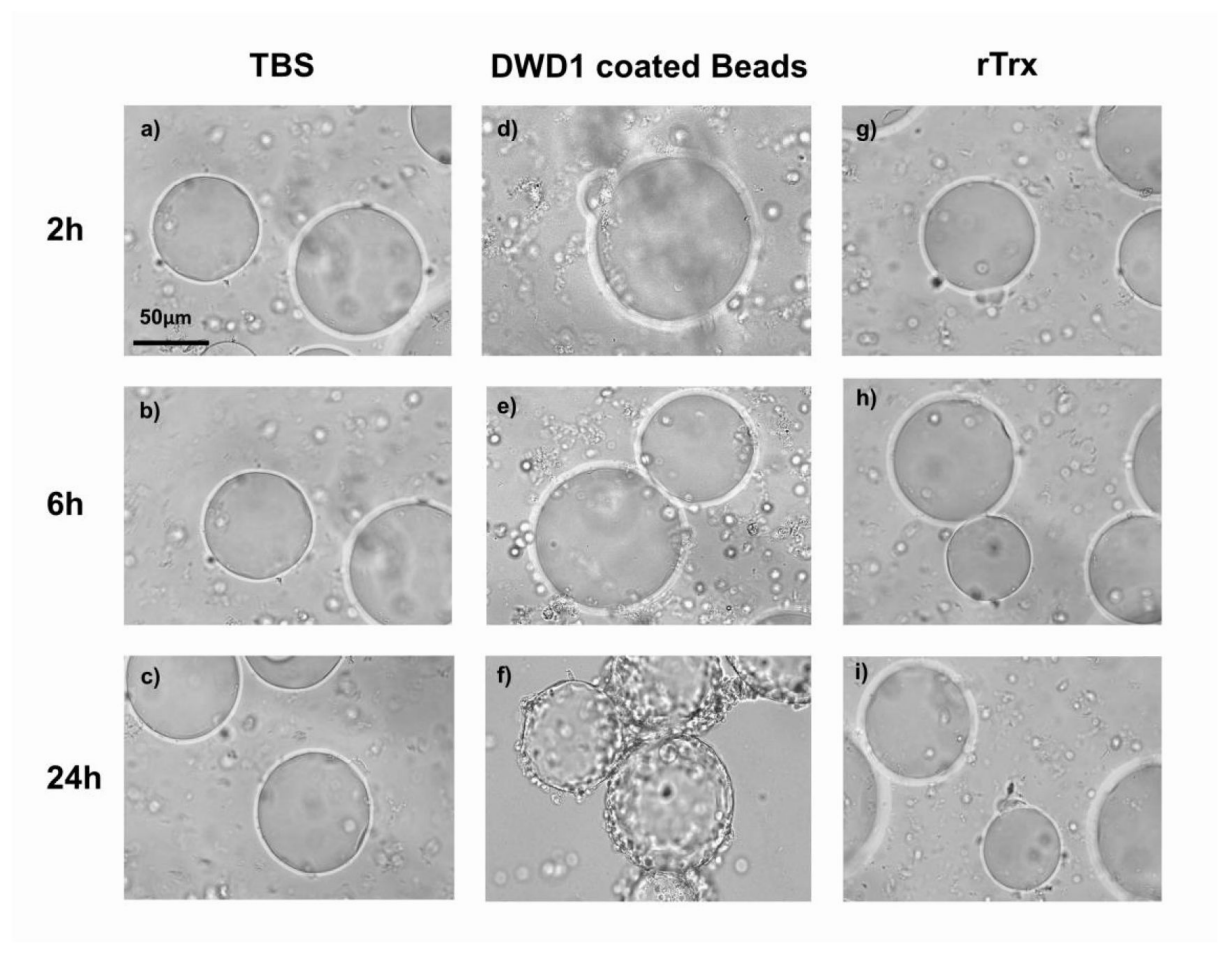
rEs-DWD1 promotes encapsulation by hemocytes. Encapsulation of protein-coated beads by hemocytes was observed by light microscopy. Beads were either not incubated with TBS (a, b, c) and rTrx (g, h, i) or coated with rEs-DWD1, followed by incubation with hemocytes for 2 h (d), 6 h (e) or 24 h (f).

An *in vitro* encapsulation assay was performed according to the described previously [43] to investigate whether rEs-DWD1 could promote cellular encapsulation. Nickel agarose beads without a protein coating were not encapsulated (Figure 9a ~ c and Figure 9g ~ i). At 2 h after incubation, only 28% of rEs-DWD1-coated beads were encapsulated (Figure 9d), while approximately 44% of the beads coated with rEs-DWD1 were encapsulated by hemocytes at 6 h (Figure 9e). Within 24 h after incubation, nearly 90% of the rEs-DWD1-coated beads were encapsulated by hemocytes (Figure 9f).

### Proteinase inhibitory activity of rEs-DWD1

**Figure 10:**
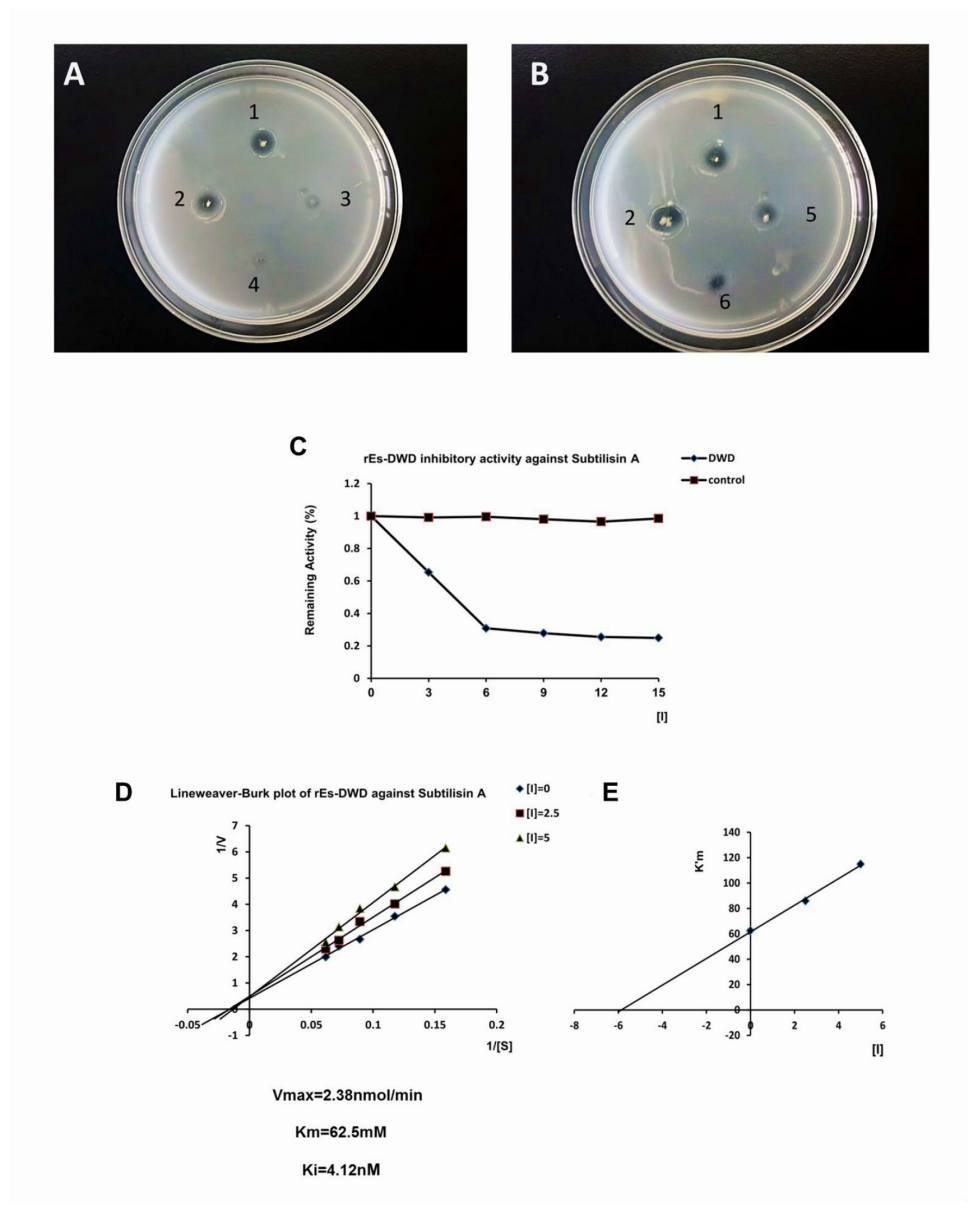
Protease inhibitory activity of rEs-DWD1. The recombinant protein rEs-DWD1 shows the result of proteinase inhibitory activity against the secretory proteinase from *B*. *subtilis*. (A) Purified rEs-DWD1. (B) Control recombinant protein expressed and purified from the *E*. *coli* expression system. 1: A single *B*. *subtilis* clone with a paper disc overlaying it. 2: A single *B*. *subtilis* clone with a paper disc containing 10 μl of 1× TBS. 3: A single *B*. *subtilis* clone with a paper disc containing 10 μl of rEs-DWD1 protein (150 μg/ml). 4: A single *B*. *subtilis* clone with a paper disc containing 10 μl of rEs-DWD1 protein (300 μg/ml). 5: A single *B*. *subtilis* clone with a paper disc containing 10 μl of rTrx (150 μg/ml). 6: A single *B*. *subtilis* clone with paper disc containing 10 μl of rTrx (300 μg/ml). (C) Inhibitory effect of rEs-DWD1 against subtilisin A. The remaining proteolytic activity on the appropriate chromogenic substrate was calculated. rTrx was a control. (D) Lineweaver–Burk plot for the competitive inhibition of subtilisin A at different concentrations: 0 μM (♦), 2.5 μM (■) and 5 μM (▲). (E) Plot of apparent K_m_ against the concentration of the inhibitor (rEs-DWD1) is shown in the inset. V_max_, K_m_ and K_i_ values are indicated underneath the graphs.

After incubation at 28° C overnight, no transparent zones formed when the purified rEs-DWD1 was added onto the paper disc (Figure 10A). By contrast, transparent zones were formed on the plate when adding TBS or rTrx onto the paper disc (Figure 10B), indicating no effect of these controls on the hydrolysis activity of protease derived from *B. subtilis*.

The potential inhibitory activity of rEs-DWD1 against subtilisin A was investigated by co-incubation of the recombinant protein with the proteinase *in vitro*. The remaining proteinase activity was determined and plotted against the concentrations of the inhibitors (Figure 10C). The rEs-DWD1 protein exhibited a strong level of inhibition on the hydrolysis of N-succinyl-Ala–Ala–Pro–Phe-p-nitroanilide (AAPF). The equilibrium dissociation constant K_i_ for the interaction of subtilisin A with rEs-DWD1 was determined directly by adding the substrate to an equilibrium mixture of proteinase and inhibitor. Lineweaver–Burk plots were constructed (Figure 10D), and the K_m_ (Figure 10E), K_i_ and V_max_ were determined to be 62.5 mM, 4.12 nM and 2.38 nmole/min, respectively.

## Discussion

Antimicrobial peptides (AMPs) as important components of endogenous antibiotic materials found in innate immune system are considered as a main element that could provide a useful way to study the innate immunity of crustaceans [44,45]. Recently, the WAP-containing protein as a source of antimicrobial peptides in crustaceans was comprehensively reviewed. The WAP domain containing proteins are a family of proteins characterized by a ‘four-disulfide-core’ (4-DSC), which perform additional functions aside from their antimicrobial activities, including as a calcium transport inhibitor in cancer metastasis [46] and an ATPase inhibitor [47,48]. Recent results reported in *Homarusgammarus* [49], *L* *. vannamei* [26] *Marsupenaeus* *japonicas* [8] and *Penaeus* *monodon* [50] have shown that they possess proteins with WAP domains exhibiting antiviral, antimicrobial and/or anti-proteinase activity [25].

In the present study, we successfully cloned the cDNA of a double WAP domain containing protein Es-DWD1. Alignment of its deduced amino acid sequences demonstrated that the EsDWD1 have two WAP domains (Figure S3A) which contain ‘four-disulfide-core’ (4-DSC) (Figure S3B) are relatively conserved. The two domains, domains I and II, in WAP proteins share limited sequence similarity both within and between species, and domain II appears to be relatively more conserved than domain I. The KxGxCP motif (including the first conserved cysteine residue) and the CxxP motif (involving the eighth conserved cysteine residue) are present in domain II but absent in domain I [4]. In the phylogenetic analysis, Es-DWD1 was grouped apart from the crustin type I and crustin type II proteins. These results indicated that Es-DWD1 is a unique molecule that arose during in the evolutionary process.

The *Es-DWD1* transcript was found to be expressed abundantly in hemocytes. The tissue expression profile of *Es-DWD1* showed similarities, especially in its high constitutive level in hemocytes. For example, *CrusEs* and *CrusEs2* from *E. sinensis* was mainly detected in hemocytes and gills [51,52]. The *MrCrs* transcripts from *Macrobrachium* *rosenbergii* were found to be expressed in hemocytes at a relatively high level. The *Fc-DWD* mRNA was detected in hemocytes and the heart at relatively high levels, but only at low levels in hepatopancreas, gills, stomach and intestine of unchallenged shrimp [25]. Altogether, these results suggested that as a component of humoral immunity, crustins are mainly expressed by circulating hemocytes. In our study, in the period of 24 h after challenge with different PAMPs, we observed acute infection symptoms in *E. sinensis* (e.g., loss of mobility) and increased mortality compared with the control group. The expression of *Es-DWD1* mRNA was upregulated significantly at different times after challenging with the various PAMPs, with peak expression levels higher than that of the control group. LPS challenge induced obviously high expression of *Es-DWD1* mRNA at 12 h. The similar expression pattern of the *SWD* transcript was also reported in *L* *. vannamei* infected with the Gram-negative bacteria *Vibrio* *alginolyticus* , which displayed an early response with a significantly increased transcript level at 3–6 h post-infection [26]. In this study, when injected with PG after 12 h, the expression of *Es-DWD1* reached up to 5 times greater than the blank control. By comparison, in the European lobster, *H* *. gammarus* , the expression level of crustin was shown to be down-regulated after challenge with the Gram-negative acteria *Listonella anguillarum* but upregulated after inoculation with the Gram-positive bacteria *Aerococcus* *viridians* var. *homari* [49]. Additionally, in *F* *. chinensis* , the expression of *Fc-DWD* was shown to increase by nearly 7-fold increase hemocytes at 12 h after viral challenge. Interestingly, the *Es-DWD1* mRNA expression showed an early response to Glu, a representative fungal PAMP, followed by rapid downregulation and then a quick rebound a peak level. Similarly, challenge with *P* *. pastoris* *GS115* in *E. sinensis* has also been found to induce a fluctuating expression pattern of *Es-DWD1* mRNA that changed from an initial downregulation to a subsequent notably high level [53]. The different responses to these different PAMPs may be due to their distinct molecular structures. Altogether, the elicitor- and time-dependent expression profiles of *Es-DWD1* reflected some degree of specificity of its response to pathogens.

Because detection of *Es-DWD1* mRNA by qRT-PCR after PAMP challenge provided insufficient evidence to make firm conclusion on its different functions as an antimicrobial peptide, we purified the rEs-DWD1 fusion protein from a prokaryotic expression system in order to test its binding affinity to Gram-negative bacteria, Gram-positive bacteria and fungus. Many WAP domain containing proteins have been reported to show different antibacterial activities [54–56]. The antimicrobial activities of rmFc-SWD against Gram-positive and Gram-negative bacteria and fungus had been displayed in *F* *. chinensis* [57]. In the spider crab ( *Hyas* *araneus* ), two isoforms of crustins have been detected containing one WAP domain in the carboxy-terminal of the mature peptide and exhibiting both antibacterial and antifungal activities [58]. In our study, the different observed affinities may due to differences in the composition of the cell walls of these microbes.

The inhibitory growth curves of the different microbes showed that the bacteria growth was inhibited by addition of rEs-DWD1 in a dose-dependent manner. As shown in Figure 9, rEs-DWD1 displayed microbial killing activities towards *E. coli* and *S. aureus* on petri dishes. In other studies, the C-type lectin AmphiCTL1 was shown to lead to cell wall damage and direct killing of microorganisms such as *S. aureus* and *S. cerevisiae* by interaction with glucans [38]. We can speculate that rEs-DWD1 may participate in a common process of antimicrobial activity. It is an inducible protein that seeks out and destroys its microbial targets, thus representing a new function for invertebrate DWD-mediated immunity [38].

We observed that rEs-DWD1 could cause the agglutination of some but not all of the microbes tested. This result indicated that the agglutination activity depends on the type of microbe targeted. Adsorption of cationic agents onto the bacterial cell membrane neutralizes and reverses the surface charge of bacteria [59], which may explain the observed bacterial agglutination [60]. In the black tiger shrimp *P* *. monodon* , rcrustinPm7 was found to induce bacterial agglutination and showed antimicrobial activity against *Vibrio harveyi*, while rcrustinPm1 could induce bacterial agglutination and exhibited antimicrobial activity against Gram-positive bacteria, *S. aureus*, *Micrococcus luteus* and *Bacillus megaterium*; however, it neither inhibited growth nor induced agglutination against Gram-negative bacteria *V. harveyi* [62]. These results are similar to our findings, suggesting that the WAP domain containing family of proteins may have a preference towards inducing agglutination of Gram-positive bacteria. Es-DWD1 molecules must crosslink bacterial cells to form the lattice involved in agglutination. Thus, adsorption of Es-DWD1 molecules can sensitize the bacterial cells to lattice formation, thereby facilitating agglutination [61]. Based on these observations, we speculate that bacterial agglutination is perhaps one mode of action for the antimicrobial activity of Es-DWD1 but not in all situations.

Results of the cellular adhesion assay showed that rEs-DWD1 functions to promote hemocyte encapsulation. We showed that attachment of rEs-DWD1 to the surface of a foreign target may be sufficient to initiate encapsulation. According to our analysis, rEs-DWD1 activated the encapsulation of the microbes by hemocytes, and the activity was more effective at 24 h than at 6 h and 2 h. The enhancement of cellular encapsulation suggested that rEs-DWD1 coating to the surface of microbes could mediate recruitment and binding of hemocytes, thereby playing a key role in the recognition and activation of intercellular interactions.

Previous reports showed that proteins containing the WAP motif exhibit protease inhibitory activities [11]. As *B. subtilis* is known to secrete proteases during growth, it is therefore capable of hydrolyzing proteins in a skim milk plate to form the characteristic transparent zones. However, no transparent zone was formed when the purified rEs-DWD1 was added, which clearly suggested a function of proteinase inhibition. In *M* *. japonicas* the Mj-DWD protein contributes to the antiviral response through its proteinase inhibitory activity. Additionally, the rFc-DWD protein found in *F* *. chinensis* can completely inhibit the activity of the proteinase(s) secreted from *B. subtilis* and *P. aeruginosa* [25]. Members of the WAP domain protein family, including SLPI and elafin, have been shown to function as serine proteinase inhibitors and perhaps to control pathogen proteinases [6]. The derived K_i_ value of rEs-DWD1 indicates that it is potentially a potent inhibitor of subtilisin A and that its primary functions are likely to include antimicrobial activities as well as to inhibit bacterial proteinases for limiting infection and pathogenesis [62].

## Concluding Remarks

In this study, we report the cloning, sequence analysis and tissue-specific distribution of the Es-DWD1 protein from *E. sinensis*. Furthermore, the binding activity, growth inhibitory activity, agglutination activity and protease inhibitory activity of the recombinant protein against bacterial and fungal microbes were assessed. Taken together, the results of our work suggest that Es-DWD1 is an inhibitor of proteinases from microorganisms and a regulator of the host-defense reactions. Thus, Es-DWD1 may be a potent immune effector that protects the crab from pathogenic infection.

## Supporting Information

Figure S1 **Multiple sequence alignment of the Es-DWD1 protein along with WAP domain containing proteins showing high similarity from a BLASTp homology search.** Identical (*) and similar (.) residues are indicated. Gaps (-) were introduced to maximize the alignment. Abbreviations: Es, *E. sinensis*; Fc, *F* *. chinensis* ; Lv, *L* *. vannamei* ; Mj, *M* *. japonicas* ; Mm, *Mus musculus*; Rn. *Rattus* *norvegicus*; Hs, *Homo sapiens*; Af, *Anoplopoma* *fimbria* ; Ss, *Salmo salar*; Om, *Oryzias* *melastigma* ; SLPI, secretory leukocyte proteinase inhibitor.
(TIF)

Figure S2 **Three-dimensional protein model of Es-DWD1.** Protein model of Es-DWD1 constructed based on a model from SWISS-MODEL. WAP domain is highlighted in dark green color. (A) Eight conserved cysteine residues (Cys^91^, Cys^102^, Cys^111^, Cys^117^, Cys^123^, Cys^124^, Cys^128^, Cys^132^) are displayed in the ball model in blue color. (B) Four disulfide bonds ((Cys^91^-Cys^124^, Cys^111^-Cys^123^ Cys^117^-Cys^132^ Cys^123^-Cys^124^)) formed between the conserved cysteine residues which are highlighted in yellow color.
(TIF)

Figure S3 **The predicted domains of Es–DWD based on amino acids sequences.** (A) The predicted double whey acid protein domains, 4DSC and signal peptides. (B) The four disulfide bridges in each domain are shown.
(TIF)

Table S1 **Primer sequences.**
(DOCX)
