# Supplementary figures and images for: A Double WAP Domain-Containing Protein Es-DWD1 from Eriocheir sinensis Exhibits Antimicrobial and Proteinase Inhibitory Activities
Source: PLoS One. 2013 Aug 13;8(8):e73563. doi: 10.1371/journal.pone.0073563 (PMC3742519; doi:10.1371/journal.pone.0073563)

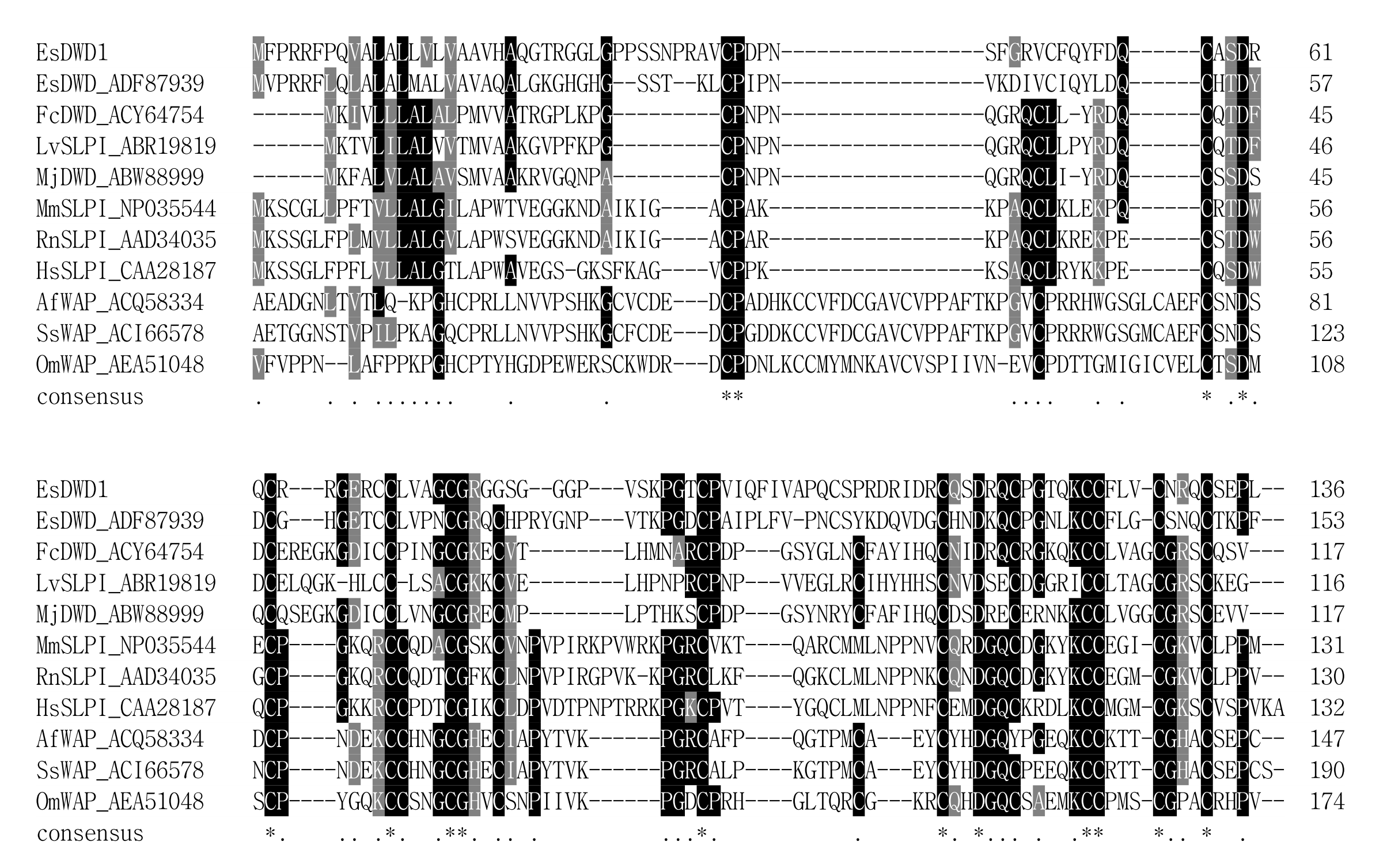

Supplement: Figure S1 — Multiple sequence alignment of the Es-DWD1 protein along with WAP domain containing proteins showing high similarity from a BLASTp homology search. Identical (*) and similar (.) residues are indicated. Gaps (-) were introduced to maximize the alignment. Abbreviations: Es, E. sinensis; Fc, F . chinensis ; Lv, L . vannamei ; Mj, M . japonicas ; Mm, Mus musculus; Rn. Rattus norvegicus; Hs, Homo sapiens; Af, Anoplopoma fimbria ; Ss, Salmo salar; Om, Oryzias melastigma ; SLPI, secretory leukocyte proteinase inhibitor. (TIF) [file pone.0073563.s001.tif]

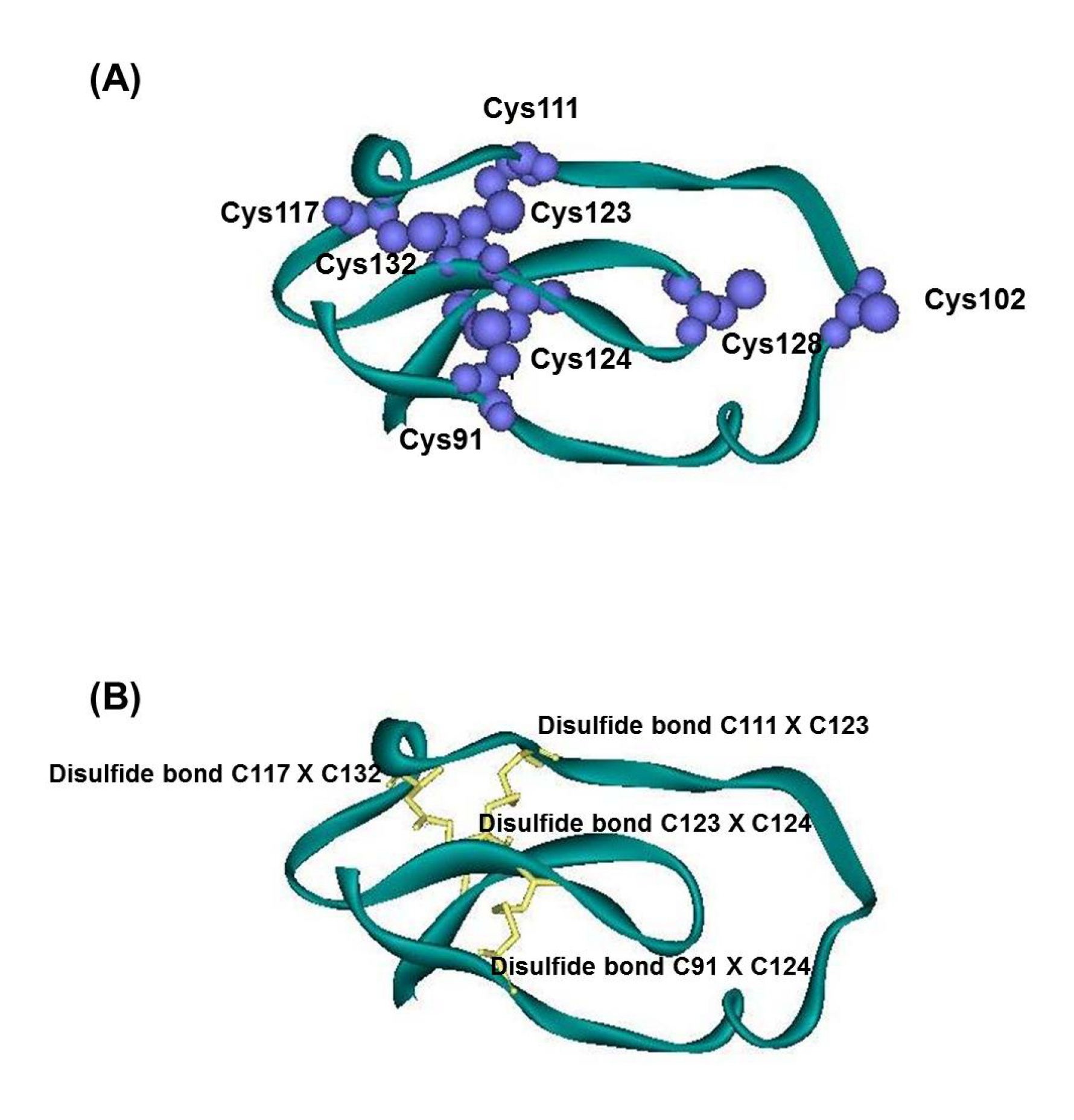

Supplement: Figure S2 — Three-dimensional protein model of Es-DWD1. Protein model of Es-DWD1 constructed based on a model from SWISS-MODEL. WAP domain is highlighted in dark green color. (A) Eight conserved cysteine residues (Cys91, Cys102, Cys111, Cys117, Cys123, Cys124, Cys128, Cys132) are displayed in the ball model in blue color. (B) Four disulfide bonds ((Cys91-Cys124, Cys111-Cys123 Cys117-Cys132 Cys123-Cys124)) formed between the conserved cysteine residues which are highlighted in yellow color. (TIF) [file pone.0073563.s002.tif]

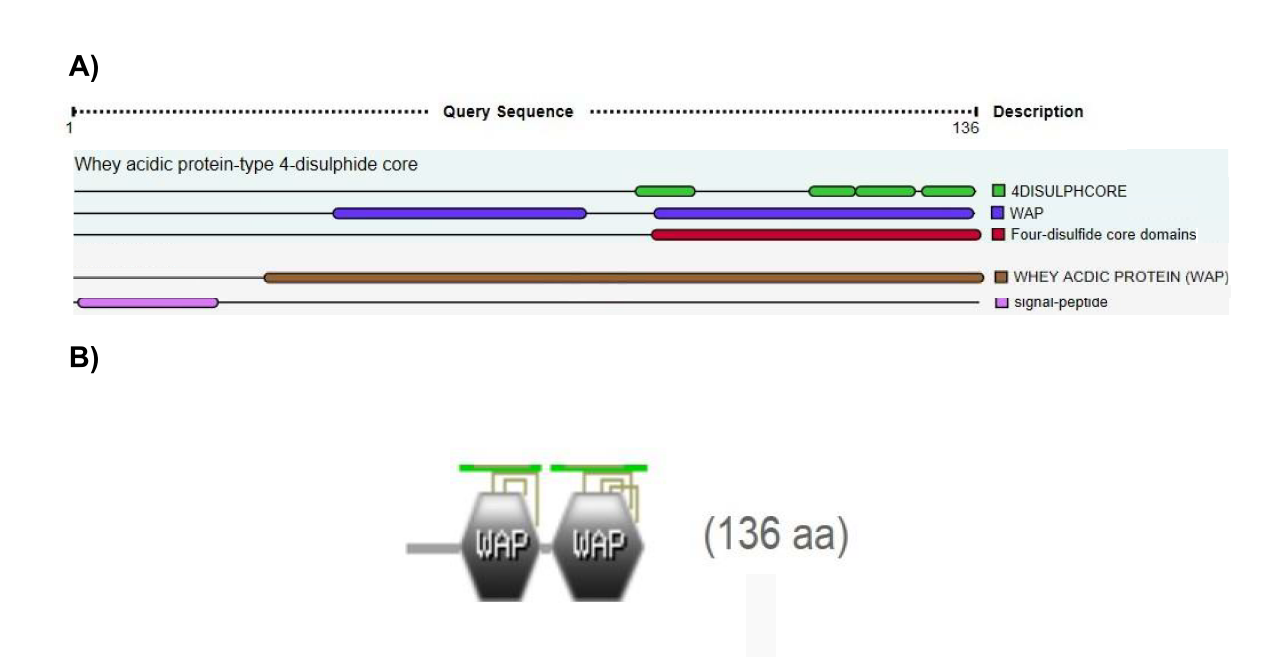

Supplement: Figure S3 — The predicted domains of Es–DWD based on amino acids sequences. (A) The predicted double whey acid protein domains, 4DSC and signal peptides. (B) The four disulfide bridges in each domain are shown. (TIF) [file pone.0073563.s003.tif]
