# Supplementary material for: A Double WAP Domain-Containing Protein Es-DWD1 from Eriocheir sinensis Exhibits Antimicrobial and Proteinase Inhibitory Activities
Source: PLoS One. 2013 Aug 13;8(8):e73563. doi: 10.1371/journal.pone.0073563 (PMC3742519; doi:10.1371/journal.pone.0073563)
Supplement: Table S1 — Primer sequences. (DOCX) [file pone.0073563.s004.docx]

**Supplemental Table S1. Primer sequences**

| **Primers by application** | **Sequence (5’-3’)** |
| --- | --- |
| ***Es-DWD1 full-length cloning*** |  |
| 5'RACE-1 | CTACAAGGACCAGGAGGGCAAG |
| 5'RACE-2 | CGATGAACTGAATGACTGGACACG |
| 5'RACE-3 | GACACACTGCCCTTGGGTTGG |
| 3'RACE-1 | TGTAGCCCTCGGGACAGAATC |
| 3'RACE-2 | GTCCAACCCAAGGGCAGTGTGTC |
| 3'RACE-3 | CGTGTCCAGTCATTCAGTTCATCGTG |
| LongUP | CTAATACGACTCACTATAGGGCAAGCAGTGGTATCAACGCAGAGT |
| ShortUP | CTAATACGACTCACTATAGGGC |
| ***qRT-PCR*** |  |
| Es-DWD1-F | CAGTGTGTCCTGACCCGAAC |
| Es-DWD1-R | AAGCAGCACTTTTGGGTC |
| β-actin-F | CTCCTGCTTGCTGATCCACATC |
| β-actin-R | GCATCCACGAGACCACTTACA |
| T7 promotor | TAATACGACTCACTATAGG |
| T7 terminator | CACCGCTGAGCAATAACTAGC |
